# Gamma-treated placental amniotic membrane allograft as the adjuvant treatment of unresponsive diabetic ulcer of the foot

**DOI:** 10.1016/j.ijscr.2019.12.033

**Published:** 2019-12-28

**Authors:** Ihsan Oesman, Witantra Dhamar Hutami

**Affiliations:** Department of Orthopaedic & Traumatology, Consultant of Foot and Ankle, Department of Orthopaedic & Traumatology, Cipto Mangunkusumo National Central Hospital and Faculty of Medicine of Universitas Indonesia, Jalan Diponegoro No. 71, Jakarta Pusat, Jakarta 10430, Indonesia

**Keywords:** Diabetic ulcer of the foot, Placental amniotic membrane, Allograft, Case series

## Abstract

**Introduction:**

Diabetic ulcer of the foot is a major cause of morbidity and is a leading cause of hospitalization in patients with diabetes, and causes productivity and financial losses that lower the quality of life of the patient. The wound is categorized as responsive and unresponsive wound, which occurs in debilitated patients as seen in diabetes mellitus. The delay in wound repair can be caused by senescent cells, absence of growth factors, and other cellular abnormalities.

**Method:**

This is a retrospective, single-centre case series with non-consecutive cases. Patients with diabetic ulcer of the foot managed using gamma-treated placental amniotic membrane, with the minimum follow up of 1 month in academic practice setting were recruited.

**Result:**

Three patients with 4 weeks period of oozing ulcers and signs of inflammation were included in this case series. Two layers of amniotic membrane dressing was applied weekly after cleaning and debridement for 3 weeks. Wound size and secretion were documented by taking photographs every week. At the end of the third week, the wound healed.

**Discussion:**

Placental amniotic membranes are composed of cells, extra-cellular matrix (ECM), and a complex of regulatory cytokines which promote cell proliferation, cell modulation, and cytokine secretion by variety of cell types involved in wound healing. Our study showed that the treatment of diabetic ulcer wound using amniotic membrane was successful in achieving wound healing in unresponsive, chronic wound of diabetic ulcer of the foot.

**Conclusion:**

Our results clearly indicated the usefulness of the application of amniotic membranes in treatment of diabetic ulcer of the foot. Amniotic membrane favoured healing of unresponsive and non-healing ulcers.

## Highlights

• Unresponsive diabetic ulcer is ulcer that does not reduce in size within a month.
• It is caused by senescent cells, absence of growth factors, and other cellular abnormalities.
• To maximize wound healing, skin substitute can be used to prevent infection and dessication.
• Amniotic membrane favoured healing of unresponsive and non-healing ulcers.

## 1 Introduction

Diabetic ulcer of the foot is a major cause of morbidity and is a leading cause of hospitalization in patients with diabetes. Diabetic ulcer of the foot can cause infection, gangrene, amputation, and even death if essential care is not provided. The rate of lower limb amputation in patients with diabetes mellitus is 15 times higher than patients without diabetes, moreover it is estimated that approximately 50 %–70 % of all lower limb amputations are due to diabetic ulcer of the foot. Moreover, diabetic ulcer of the foot can cause significant emotional and physical distress as well as productivity and financial losses that lower the quality of life of the patient [1].

The cause of diabetic ulcer of the foot is multifactorial, however, the main contributing factors are peripheral neuropathy and peripheral vascular disease. The neuropathy is caused by hyperglycemia-induced metabolic abnormalities. The condition of hyperglycemia causes an increase in the action of anzyme aldose reductase and sorbitol dehydrogenase, causing the conversion of intracellular glucose to sorbitol and fructose. The accumulation of sorbitol and fructose will cause a decrease in the synthesis of nerve cell myoinositol required for normal nerve conduction. Moreover, the conversion of glucose will result in depletion of store of nicotinamide adenine dinucleotide phosphate, which is essential for the detoxification of reactive oxygen species (ROS) and for the synthesis of the vasodilator nitric oxide. The neuropathy in diabetic ulcer includes the motor, autonomic, and sensoric components. Loss of sensation causes patient to be unable to detect the injury occured on lower extremity. Autonomic neuropathy causes diminished sweat and oil gland, causing the foot to lose its natural ability to moisture the skin, and therefore the skin is increasingly susceptible to tears and subsequent infection. Persistent hyperglycemia causes endothelial cell dysfunction and smooth cell abnormality in peripheral arteries, with the resultant of vasoconstriction and its subsequent ischemia of the lower limb [2].

The cellular mechanism in wound repair is categorized into inflammatory, proliferative (granulation phase), and remodelling phase. In healthy individual, intrinsic medical conditions nor extrinsic environmental factors rarely affect the repair process. In contrast to wound healing in healthy individual, the phases of repair in chronic wound does not follow the normal sequence of healing. The wound is categorized as responsive and unresponsive wound, which occurs in debilitated patients as seen in diabetes mellitus. This unresponsive wound must be treated based on triad of care consisting of intrinsic, extrinsic, and wound environment factors. Unresponsive diabetic ulcer of the foot is defined as ulcer that does not show a reduction in size in one-month period of adequate treatment. The delay in wound repair can be caused by senescent cells, absence of growth factors, and other cellular abnormalities. Senescent cells are defined as viable cells, but have lost their ability to proliferate. This cause these cells to be unresponsive to endogenous and exogenous growth factorbin wound milieu. The wound environment factor includes wound bed status, cellular activity of the wound, and devices and dressing used for the treatment. About 15–20 % of chronic wound does not respond to conventional therapy and requires advanced technologies to stimulate the tissue repair. Debridement of senescent cells and nonviable tissue will increase the availability of viable cells to produce and respond to growth factors and other cytokines [3].

To maximize the healing of wound, skin substitute can be used to prevent infection and dessication. This temporary skin coverage can be provided by synthetic or biological dressings. The biological dressing includes placental amniotic membrane, allograft skin, or xenograft skin. Compared to other biological dressing, placental amniotic membrane has advantages as ideal dressing. Placental amniotic membrane can act as an effective barrier, reduces loss of heat, fluid, and protein, and has a good adherence to the wound. It also has bacteriostatic property, therefore reduces the incidence of infection. Moreover, it has some analgesic effect, and importantly has no immunological reaction [4]. Histologically, amniotic membrane is similar to the skin. Its inner layer is composed on cuboidal cells, whereas the outer layer is composed of mesenchymal connective tissue. It has no blood vessel or lymphatic channel. The aim of radiation in the preparation of amniotic membrane is to prolong its shelf-life [4].

In Indonesia, preparation of placental amniotic membrane has been available commercially, provided by BATAN (National Nuclear Energy Agency) Research Tissue Bank in Jakarta. Started on 1986, BATAN Research Tissue Bank has developed the preservation of fresh amniotic membrane by lyophilization and sterilization by gamma radiation. The fresh placental amniotic membranes contain collagen, hormones, and enzymes and is obtained from fetal placenta delivered from healthy mothers who are free form diseases including human immunodeficiency (HIV) infection, and hepatitis B and C [5].

We presented a case series of diabetic ulcer patients treated with gamma-treated placental amniotic membrane allograft. This case series had been presented in line with PROCESS guideline [6].

## 2 Method

This is a retrospective, single-centre case series with non-consecutive cases. Patients with diabetic ulcer of the foot managed in academic practice setting were recruited. Patients were treated in Dr. Cipto Mangunkusumo Hospital Jakarta during December 2018. The exposure was debridement and treatment using gamma-treated placental amniotic membrane, with the minimum follow up of 1 month. The data was collected using medical record. The inclusion criteria for study participant was adult with diabetic ulcer and the exclusion criteria was other immunocompromised conditions.

**Fig. 1:**
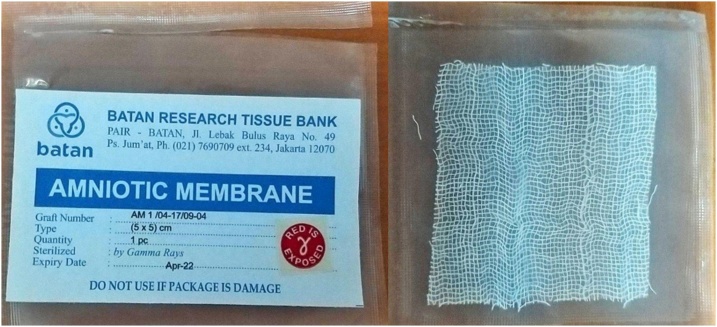
Amniotic Membrane Preparation.

After placental amniotic membranes from the screened donor were selected, the membrane were collected and prepared. The preparation of placenta was started with washing the fresh amniotic membrane using sterile saline followed by washing in 0.05 % sodium hypochlorite solution and then sterile distilled water until they were completely cleared of blood particles. Finally the membrane were sterilized by gamma irradiatin 25 kGy [4]. This amniotic membrane was prepared in BATAN Research Tissue Bank, Jakarta (Fig. 1).

## 3 Result

**Fig. 2:**
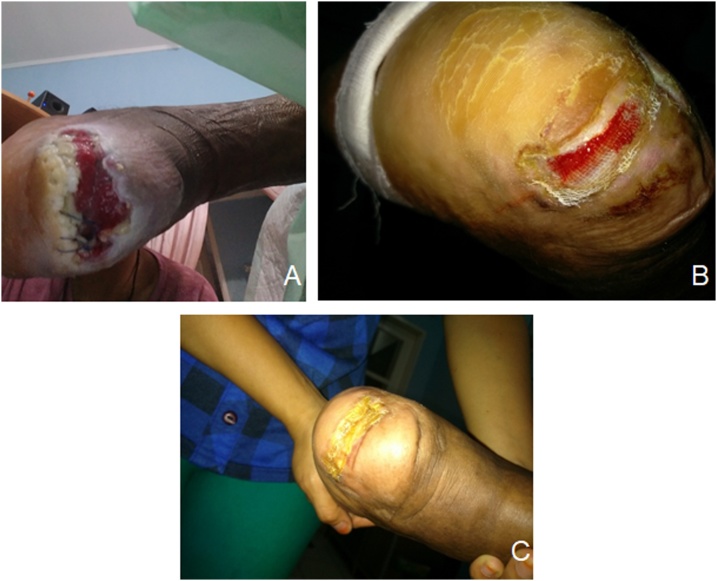
Clinical Manifestation of Patient One.

**Fig. 3:**
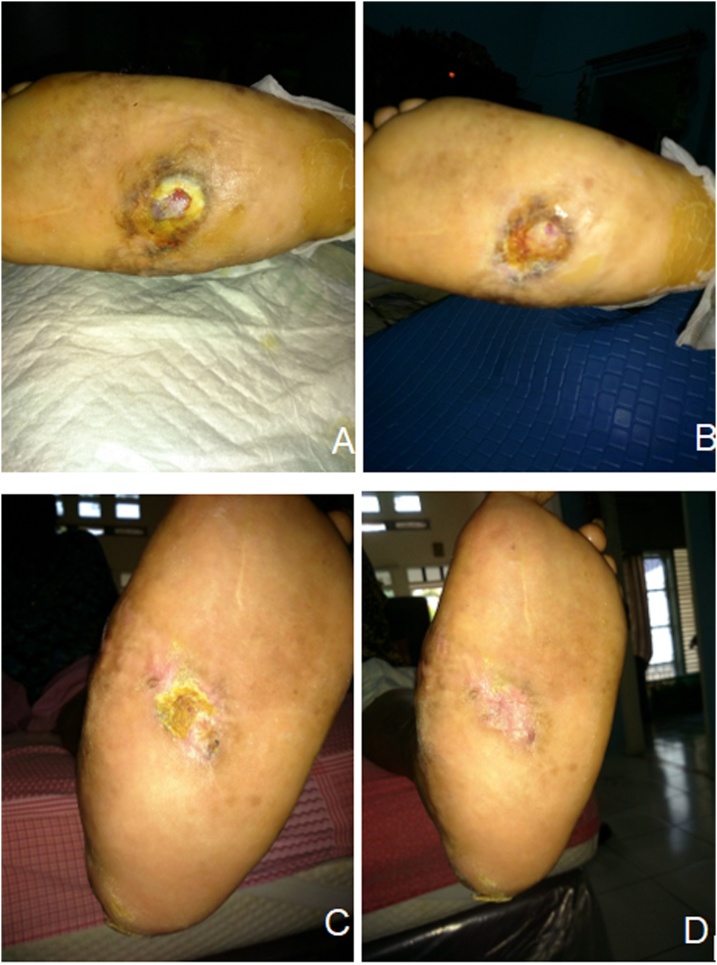
Clinical Manifestation of Patient Two.

**Fig. 4:**
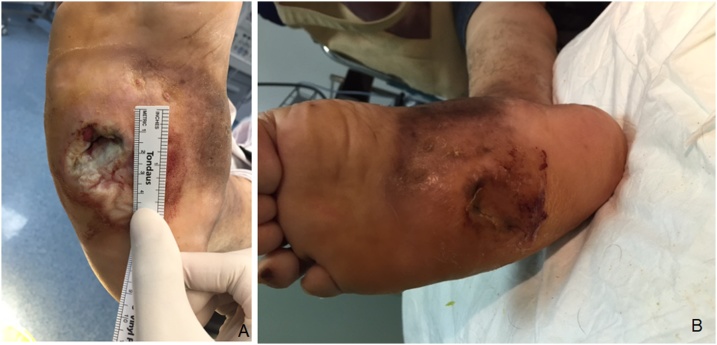
Clinical Manifestation of Patient Four.

Three patients were included in this case series. The baseline data of the patient is described in Table 1. Patients came with history of trauma, and after 4 weeks of nonoperative treatment, the pain had not subsided. Clinical examination showed oozing ulcer in all three patients with signs of inflammation. No exposed bone was found on examination. Before application of amniotic membrane, the wound of each patient was measured and cleaned. Two layers of amniotic membrane dressing was applied weekly after cleaning and debridement for 3 weeks. Standard dressing was then applied over the graft. The patient was advised not to remove the dressing and to keep it dry until the next visit. Wound size and secretion were documented by taking photographs every week (Figs. 2–4). The debridement and dressing was performed by the single surgeon (I.O).

**Table 1:** Baseline Data of the Patient.

No	Sex	Age (years)	Time DM Diagnosis	Complaint
1	M	54	2 years	Pain and oozing, unhealing ulcer
2	M	63	3 years	Pain and oozing, unhealing ulcer
3	F	48	2.5 years	Pain and oozing, unhealing ulcer

In each week, the size of the ulcer was decreasing, the pus diminished, and reepithelialisation occurred. Pain also diminished. At the end of the third week, the wound healed with no ulcer appeared. The signs of inflammation also disappeared.

## 4 Discussion

Placental amniotic membranes are composed of cells, extra-cellular matrix (ECM), and a complex of regulatory cytokines which function to support tissue growth and modulate inflammation. They also promote cell proliferation and cell modulation, and modulate cytokine secretion by variety of cell types involved in wound healing. There are about 226 growth factors identified in amniotic membrane, including growth factors, cytokines, chemokines, and regulatory inhibitors of metalloproteinases (TIMPs), such as PDGF‐AA, PDGF‐BB, TGF‐α, TGF‐β, bFGF, EGF, VEGF, IL‐10, IL‐4, placental growth factor (PlGF), TIMP‐ 1, TIMP‐2 and TIMP‐4, which possess important regulatory roles in regulating fetal development and pregnancy.

Fetal placental tissues contain low levels of HLA antigens and has no immune rejection. Therefore, placental tissues can be cleansed to remove blood and hazardous materials, while preserving the natural biological activity of the tissue for trans‐ plantation without complete decellularization. To provide a product for patient use, allograft tissues require preservation techniques to allow for transportation and storage. The most common method to preserve tissue grafts and prevent degradation is through cryopreservation or freezing. An increasingly popular alternative to cryopreservation is tissue dehydration. Tissue can be dehydrated under heat, open air, or freeze drying (lyophilization). By removing residual moisture, tissue can be preserved by reducing activity of soluble chemical reactions and water‐ dependent enzymatic activity and inhibiting the viability of microorganisms in a low moisture environment. Dehydration preserves tissue without the need for freezers, dry ice, or liquid nitrogen and certain methods of dehydration have been shown to retain equivalent or superior biological activity compared to cryopreservation, with the benefits of being shipped and stored at ambient conditions. Dehydrated tissues are also typically stronger and easier to handle than wet tissues. Though dehydration may alter the tissue's microstructure by causing the matrix to become more compact in the absence of water, by preserving the native tissue matrix proteins the dehydrated tissue can be rehydrated in the wound environment to return the tissue to its original state. Following dehydration, human amniotic membrane tissue is easy to handle and can be stored at ambient conditions with a shelf‐life of up to 5 years, while preserving the structural integrity and biochemical activity of native amniotic membrane. Even though dehydration renders amniotic cells nonviable, these cells remain structurally intact, including the cellular and pericellular components that play essential roles in regulating biological activity. Retention of bioactive factors is thought to be critical to the clinical efficacy of amniotic tissue allografts in wound repair and tissue regeneration. Therefore, harsh cleansing processes that wash bioactive material out of the grafts may greatly reduce the cytokine content and diminish the clinical efficacy of the naturally derived tissues. An additional benefit of tissue dehydration is that the allografts can be terminally sterilized to reduce the risk of infectious disease from the donor tissue. While all allograft tissues are aseptically processed to reduce the risk of bacterial or viral contamination, dehydrated tissues can be terminally sterilized using techniques such as gamma ray or electron beam irradiation to further reduce the risk of disease transmission. Though high levels of radiation may potentially crosslink or denature proteins within tissues, terminally sterilized amniotic membranes allografts have been proven to retain biological activity both clinically and through in vitro experiments. These data suggest that sterilization does not significantly diminish the bioactivity of amniotic membrane allografts and is worthwhile to ensure maximal safety to patients [4].

Our study showed that the treatment of diabetic ulcer wound using amniotic membrane was successful achieving wound healing. This is similar to study by Barski et al. and ElHeneidy et al. who found that the application of amniotic membrane help in wound healing [7,8]. In meta-analysis of wound care in diabetic foot performed by Loffelbeil et al. [9], they found that wound care using amniotic membrane resulted in less hypertrophic scarring when compared to wound without amniotic membrane. This finding supported our finding that amniotic membrane graft is effective in accelerating healing of diabetic foot ulcer. In the study by Loffelbeil et al. [9] in the use of amniotic membrane for wound care in swine and human models, they found that in the swine model, the use of amniotic membrane prevented cicatrisation and enhanced the formation of basement membrane. The enhanced formation of basement membrane will improve defensive capacities of the wound against microbial infections, because the basement membrane forms a line of resistance, even if the overlying epithelial layer is not complete. The advantage of amniotic membrane dressing in human model was statistically significant, and can be used as cost effective alternative of wound dressing particularly in developing country [9]. The findings in the previous studies support the finding in our case series, in which the use of placental amniotic membrane improved healing in unresponsive diabetic ulcer of the foot and that it has the potency of being cost effective in the developing country like Indonesia.

The strength of our study was that the intervention given between the patients was similar to each other and that this treatment provided new insight in the treatment of unhealed or unresponsive diabetic ulcer of the foot. However, our study size was small. A larger, better study in the future is awaited.

## 5 Conclusion

Our results clearly indicated the usefulness of the application of amniotic membranes in treatment of diabetic ulcer of the foot. Amniotic membrane improved healing of unresponsive and non-healing ulcers.

## Conflict of interest

The authors certify that they have NO affiliations with or involvement in any organization or entity with any financial interest or non-financial interest in the subject matter or materials discussed in this manuscript.

## Sources of funding

The authors received no financial support for the research, authorship, and/or publication of this article.

## Ethical approval

The ethical approval was provided.

## Consent

Informed consent had been obtained from the patient before the manuscript was written.

## Author contribution

Ihsan Oesman: study concept, data collection, data interpretation, and writing the paper.

Witantra Dhamar Hutami: data collection, data interpretation and writing the paper.

## Registration of research studies

This case series is registered in ANZCTR (Australian New Zealand Clinical Trials Registry) with the trial number of 378871.

## Guarantor

Ihsan Oesman.

## Provenance and peer review

Not commissioned, externally peer-reviewed
